# Characterization of Expanded Gamma Delta T Cells from Atypical X-SCID Patient Reveals Preserved Function and IL2RG-Mediated Signaling

**DOI:** 10.1007/s10875-022-01375-6

**Published:** 2022-10-19

**Authors:** Elina A. Tuovinen, Sakari Pöysti, Firas Hamdan, Kim My Le, Salla Keskitalo, Tanja Turunen, Léa Minier, Nanni Mamia, Kaarina Heiskanen, Markku Varjosalo, Vincenzo Cerullo, Juha Kere, Mikko R. J. Seppänen, Arno Hänninen, Juha Grönholm

**Affiliations:** 1 Translational Immunology Research Program, University of Helsinki, Helsinki, Finland; 2 Folkhälsan Research Center, Helsinki, Finland; 3 Pediatric Research Center, New Children’s Hospital, University of Helsinki and HUS Helsinki University Hospital, Helsinki, Finland; 4 Department of Clinical Microbiology and Immunology, Turku University Hospital, Turku, Finland; 5 Drug Research Program Helsinki (DRP), Faculty of Pharmacy, University of Helsinki, Helsinki, Finland; 6 Digital Precision Cancer Medicine Flagship (iCAN), University of Helsinki, Helsinki, Finland; 7 Systems Biology Research Group and Proteomics Unit, Institute of Biotechnology, HiLIFE, University of Helsinki, Helsinki, Finland; 8 Faculty of Science and Technology, University of Lille, Lille, France; 9 Children’s Immunodeficiency Unit, New Children’s Hospital, University of Helsinki and HUS Helsinki University Hospital, Helsinki, Finland; 10 Department of Biosciences and Nutrition, Karolinska Institutet, Stockholm, Sweden; 11 Stem Cells and Metabolism Research Program, University of Helsinki, Helsinki, Finland; 12 Rare Diseases Center and Pediatric Research Center, New Children’s Hospital, University of Helsinki and HUS Helsinki University Hospital, Helsinki, Finland

**Keywords:** X-linked combined immunodeficiency, Severe combined immunodeficiency, atypical, Interleukin receptor common subunit gamma, IL2RG, Gamma-delta T-cell receptor

## Abstract

Abnormally high γδ T cell numbers among individuals with atypical SCID have been reported but detailed immunophenotyping and functional characterization of these expanded γδ T cells are limited. We have previously reported atypical SCID phenotype caused by hypomorphic *IL2RG* (NM_000206.3) c.172C > T;p.(Pro58Ser) variant. Here, we have further investigated the index patient’s abnormally large γδ T cell population in terms of function and phenotype by studying IL2RG cell surface expression, STAT tyrosine phosphorylation and blast formation in response to interleukin stimulation, immunophenotyping, TCRvγ sequencing, and target cell killing. In contrast to his ⍺β T cells, the patient’s γδ T cells showed normal IL2RG cell surface expression and normal or enhanced IL2RG-mediated signaling. Vδ2 + population was proportionally increased with a preponderance of memory phenotypes and high overall tendency towards perforin expression. The patient’s γδ T cells showed enhanced cytotoxicity towards A549 cancer cells. His TCRvγ repertoire was versatile but sequencing of *IL2RG* revealed a novel c.534C > A; p.(Phe178Leu) somatic missense variant restricted to γδ T cells. Over time this variant became predominant in γδ T cells, though initially present only in part of them. IL2RG-Pro58Ser/Phe178Leu variant showed higher cell surface expression compared to IL2RG-Pro58Ser variant in stable HEK293 cell lines, suggesting that somatic p.(Phe178Leu) variant may at least partially rescue the pathogenic effect of germline p.(Pro58Ser) variant. In conclusion, our report indicates that expansion of γδ T cells associated with atypical SCID needs further studying and cannot exclusively be deemed as a homeostatic response to low numbers of conventional T cells.

**Supplementary Information:**

The online version contains supplementary material available at 10.1007/s10875-022-01375-6.

## Introduction

Severe combined immunodeficiencies (SCID) are genetic disorders caused by disease-causing variants in genes regulating lymphocyte differentiation and proliferation [1]. Typical SCID is defined by very low or undetectable numbers of CD3 + T cells (< 300/μl) [2, 3] and, depending on the causative gene, functionally deficient or absent NK cells and/or B lymphocytes [1]. Without stem cell transplantation or gene therapy, SCID patients succumb to infections within the first 2 years of life [4, 5]. Recently, growing numbers of hypomorphic variants in genes causing SCID have been described [6–17]. These present with milder clinical phenotype, with partially preserved T cell number and/or function. Thus, this condition is often coined as “leaky” or atypical SCID [3]. There are no universally accepted diagnostic criteria for atypical SCID. Phenotypically, these patients commonly display reduced or declining numbers of T cells and/or their subsets, and impaired T cell function [2, 3]. In some cases, increased proportions of γδ T cells may lead to normal absolute CD3 + T cell counts [17–22]. Lack of cytomegalovirus (CMV) infection in some patients, a known driver for γδ T cell expansion [20, 23], suggests yet undiscovered factors elevating γδ T cell counts [20].

γδ T cells are the first T cells to appear during thymic development [24] and normally comprise ca. 1–10% of peripheral blood CD3 + T lymphocytes [25, 26]. They are present at higher frequencies in epithelial barriers of the skin, intestines, and lungs, offering the first line of defense as rapid responders to various types of antigens and stimuli in a major histocompatibility complex (MHC) independent manner [24, 27]. γδ T cells act as early producers of cytokines like interferon (IFN)-γ and interleukin (IL)-4 [24] and as antigen presenting cells and thus have attributes of both the innate and adaptive immunity [27–29]. Most γδ T cells in the peripheral blood present Vγ9Vδ2 T cell receptor (TCR) [27, 30]. CMV infection, however, does not affect the major Vγ9Vδ2 + population but rather the Vδ1 + and Vδ3 + populations [31, 32]. In addition to host defense functions, γδ T cells play important roles in tissue homeostasis and tumorigenesis [31]. Though protumor roles have been reported, γδ T cells are mainly investigated for their potential in immunotherapeutic applications for cancer [33, 34].

We have previously described a patient with novel c.172C > T;p.(Pro58Ser) variant in IL-2 receptor gamma chain (*IL2RG*; NM_000206.3) associated with atypical X-SCID phenotype and abnormally large peripheral blood γδ T cell number comprising of up to 37.3% of CD3 + lymphocytes [17]. Here, we have further studied the phenotype and function of these γδ T cells presenting with normal IL2RG surface expression, IL2RG-dependent signaling, and normal or enhanced killing of *in vitro* target cells. In addition, we discovered a single base pair somatic c.534C > A; p.(Phe178Leu) *IL2RG* variant restricted to his γδ T cells. We studied the effect of this somatic variant with BioID proximity labeling in IL2RG expressing HEK293 cell lines and found that it altered protein–protein interactions of IL2RG-Pro58Ser. We also noted higher cell surface expression of IL2RG-Pro58Ser/Phe178Leu variant compared to IL2RG-Pro58Ser variant alone in these HEK293 cell lines, suggesting that the somatic variant may at least partially rescue the effect of Pro58Ser variant.

## Materials and Methods

### Genetic Analyses, Capillary Sequencing, and RT-PCR

Capillary sequencing methods have been described elsewhere [17]. Cell isolation is described in the Online Resource. TCRγ sequencing on complementarity-determining region 3 (CDR3) was performed according to the manufacturer’s instructions on genomic DNA (gDNA) isolated from γδ cells with TCRG immunoSEQ assay (Adaptive Biotechnologies), described in more detail in the Online Resource. RNA was isolated with Nucleospin RNA isolation kit (Macherey–Nagel) according to the manufacturer’s instructions. All available RNA was used as template for cDNA synthesis (BioRAd iScript cDNA synthesis kit; according to the manufacturer’s instructions). Primer sequencies are listed in Table S1, Online Resource.

### Flow Cytometry and Antibodies

Flow cytometry was performed on both whole blood and peripheral blood mononuclear cells (PBMCs). Staining was performed with fluorescent-conjugated antibodies and is described in more detail in the Online Resource. To measure signal transducer and activator of transcription (STAT) 5 tyrosine phosphorylation, isolated PBMCs were stimulated with IL-2 (10 U/ml and 320 U/ml, R&D Systems), IL-7 (50 ng/ml, Miltenyi Biotec), or IL-15 (6 ng/ml and 100 ng/ml, R&D Systems). For STAT3 and STAT6 tyrosine phosphorylation, PBMCs were stimulated with 50 ng/ml IL-21 (Peprotech) or with 100 ng/ml IL-4 (Biotechne), respectively. Blast formation of TCR αβ and γδ cells was determined by flow-cytometric assay for specific cell-mediated immune-responses in activated whole blood (FASCIA) [35]. To study CD25 and CD69 upregulation in response to TCR stimulation and intracellular cytokine expression, PBMCs were stimulated with plate bound anti-CD3/28 or Biolegend’s Activation cocktail (phorbol 12-myristate-13-acetate/ionomycin with Prefeldin A), respectively. For γδ T cell phenotyping, patient and healthy donor PBMCs were either directly stained to determine the baseline or cultured for 24 h in low glucose RPMI medium supplemented with 10% FBS, 2 mM L-glutamine, and 100 IU/ml penicillin–streptomycin with three different stimuli: (1) 1 mg/ml of anti-CD3 and anti-CD28; (2) 5 mM Zoledronic acid (Sigma) and 1000 IU/ml of human IL-2 (R&D Systems); (3) 5 mM Zoledronic acid. Cells were then incubated with Fc-block (Biolegend) and labelled with fluorescent-conjugated antibodies for CdVd1, CdVd2, CD25, CD69, CD45RA, CD62L, CD3, CD4, CD8, Perforin, CD27, and FasL. Cells were analyzed with BD Fortessa flow cytometer and FlowJo (v.10.7.2) software. All monoclonal antibodies and their details are listed in Table S2, Online Resource. All the protocols are described in detail in the Online Resource.

### LDH Release Assay

Killing assays are described in detail in the Online Resource. Briefly, target cells to determine effector cell–mediated cell lysis were A549 (human adenocarcinoma), MDA-MB-436 (human triple breast cancer), and Daudi cells (human Burkitt lymphoma). Freshly isolated PBMCs or γδ T cells were added at a 1:40 or 1:20 (target to effector) ratio, respectively, and incubated for 4 h at + 37 °C followed by supernatant collection. Cell lysis was determined by lactate dehydrogenase (LDH) releases using the CyQUANT LDH Cytotoxicity assay kit (Invitrogen) according to the manufacturer’s instructions.

### IL2RG Expressing Flp-In 293 T-REx Cell Lines and Proteomics

In silico computational analysis and plasmid mutagenesis are described in the Online Resource. Generation of the inducible HEK293 Flp-In Trex cell lines expressing C-terminally MAC-tagged IL2RG constructs and the sample preparation for mass spectrometry have been described elsewhere [36, 37] and in detail in the Online Resource together with proteomic analysis and mass spectrometry data processing. Cell surface and total cell-associated IL2RG expression levels in these HEK293 Flp-In Trex cell lines were analyzed by flow cytometry and are described in the Online Resource.

## Results

### Patient with Hypomorphic IL2RG p.(Pro58Ser) Variant Has Elevated Proportion of γδ T Cells in His Peripheral Blood

**Fig. 1:**
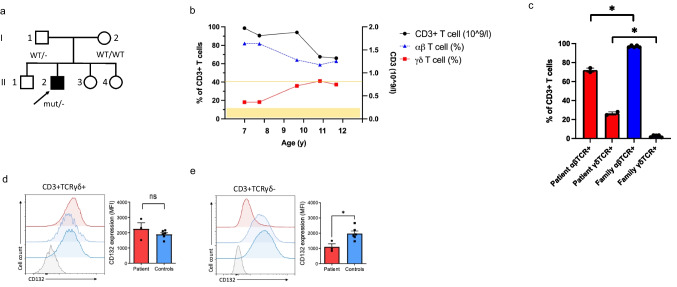
Patient’s γδ T cells show normal surface expression of IL2RG. **a** Family pedigree. Index patient marked with an arrow, WT for wild type. **b** Patient’s total CD3 + T cell levels and percentual proportions of TCRαβ + and TCRγδ + cells of peripheral blood CD3 + T cells over time. Yellow area indicates normal range of the γδ T cells (1.9–11.7%; in-house reference range by HUSLAB), yellow line indicates the lower limit of normal CD3 + T cell absolute number (0.75 × 10^9/l; in-house reference range by HUSLAB). **c** TCRαβ + and TCRγδ + proportions of the patient and his family members (mean with SEM). Cell surface expression of IL2RG (CD132) in CD3 + TCRγδ + (**d**) and CD3 + TCRγδ- (**e**) cells. Patient in red, healthy donor 1 in light blue, healthy donor 2 in blue, fluorescent minus one sample in gray. Shown are representative histograms (left) and cumulative bar graphs from three independent experiments (for healthy controls *n* = 2 in each repeat). Graphs show mean with SEM. *p < 0.05, determined by unpaired *t*-test with Welch’s correction; ns, non-significant in (**c**–**e**). The figure was constructed using the following software: Microsoft Office Powerpoint (64B), GraphPad Prism (v.9.2.0), NovoExpress software, Acea

Our previously published index patient with novel hemizygous *IL2RG* c.172C > T;p.(Pro58Ser) missense variant [17] is currently 13-year-old male (Fig. 1a). At age 2, he started to suffer from recurrent respiratory tract infections and persistent bilateral purulent middle ear infections. He developed bronchiectasis by the age of 7. Despite normal total immunoglobulin levels, intravenous immunoglobulin (IVIG) treatment was started due to specific antibody deficiency and recurrent respiratory tract infections. Thereafter, frequency of infections has markedly reduced and no progression of bronchiectasis has been observed. Before immunoglobulin substitution, our patient’s PCR test for CMV was negative. After the start of IVIG, our patient has repeatedly tested positive for CMV-specific IgG, likely due to IVIG, but has remained negative for IgM CMV antibodies. Currently, he suffers from molluscum contagiosum with occasional secondary bacterial skin infections. His detailed clinical phenotype and genetic analysis have been described earlier [17] and were updated in the Online Resource (text and Table S3). Since the age of 7, he has showed increased amounts of γδ T cells in his peripheral blood (20–40% of CD3 + T cells), unlike his family members (Fig. 1b, c, Fig. S1a, Online Resource), while the total amount of CD3 + T cells has remained within normal range (Fig. 1b, Table S3 Online Resource). The presence of c.172C > T p.(Pro58Ser) missense variant in γδ T cells was verified by Sanger sequencing, ruling out genetic reversion (Fig. S1b, Online Resource). Expression of IL2RG p.(Pro58Ser) variant was reduced on the surface of CD4 + and CD8 + T cells and NK cells, as well as on monocytes [17]. We studied the surface expression of IL2RG (CD132) on the patient’s γδ T cells and found it to be normal when compared to healthy donors’ cells while being clearly reduced on his ⍺β T cells (CD3 + TCRγδ- cells) (Fig. 1d, e). However, the total cell-associated IL2RG was significantly reduced also in his γδ T cells (34% less than in controls), but reduction of total IL2RG expression was even more pronounced in his TCRγδ-negative T cells (51% less than in controls, Fig. S2a-b, Online Resource). Taken together, IL2RG p.(Pro58Ser) variant leads to reduced IL2RG cell surface expression on conventional CD3 + T cells, while γδ T cells express normal surface level of IL2RG.

### IL-2, IL-4, IL-7, IL-15, and IL-21 Signaling Is Intact in Patient’s γδ T Cells, While Selectively Impaired in His ⍺β T Cells

**Fig. 2:**
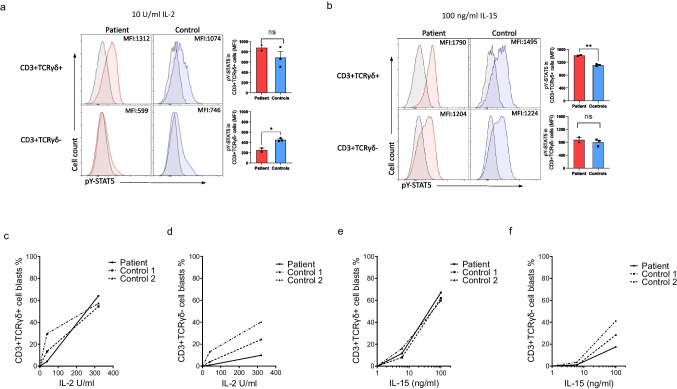
IL2RG-mediated IL-2 and IL-15 signaling is intact in patient’s γδ T cells. STAT5 phosphorylation in response to 10 U/ml IL-2 (**a**) and 100 ng/ml IL-15 (**b**). On the left are shown representative histograms and on the right are shown cumulative histograms from two independent experiments (for healthy controls *n* = 3, MFI determined stimulated — unstimulated). **c**–**f** Patient’s and healthy donors’ T cell blast formation determined by FASCIA. CD3 + TCRγδ + (**c**) and CD3 + TCRγδ- cells (**d**) stimulated with IL-2, CD3 + TCRγδ + (**e**) and CD3 + TCRγδ- cells **(f**) stimulated with IL-15 (solid line for patient, dotted line for the controls). Data are representative of two different experiments. *p < 0.05; **p < 0.01, determined by unpaired *t*-test with Welch’s correction; ns, non-significant in (**a**–**b**). The figure was constructed using the following software: Microsoft Office Powerpoint (64B), NovoExpress software, Acea

To study IL2RG downstream signaling in T cells, we measured STAT5 tyrosine phosphorylation (pY-STAT5) in response to IL-2 stimulation by flow. We found that pY-STAT5 was significantly reduced in the patient’s TCRγδ-negative CD3 + T cells (representing ⍺β T cells), while it was normal in his TCRγδ-positive CD3 + T cells (Fig. 2a). Next, we evaluated pY-STAT5 in response to IL-15 stimulation and found that the patient’s γδ T cells showed slightly increased pY-STAT5 compared to healthy donors’ γδ T cells, while there was no difference observed in TCRγδ-negative T cells (Fig. 2b). Furthermore, we observed no clear difference in IL-4-induced STAT6 tyrosine phosphorylation, IL-21-induced STAT3 tyrosine phosphorylation, or IL-7-induced STAT5 tyrosine phosphorylation between healthy donors’ and the patient’s γδ or αβ T cells (Fig. S3a-c, Online Resource).

With FASCIA [35], the patient’s γδ T cells showed slightly reduced blast formation in response to low dose of IL-2 (10 U/ml) but with high dose of IL-2 (320 U/ml) it was comparable to controls, while blast formation of the patient’s ⍺β T cells remained low with both doses (Fig. 2c, d). Blast formation in response to IL-15 was comparable between patient and healthy donor γδ T cells, while it was reduced in response to high dose of IL-15 (100 ng/ml) in the patient’s ⍺β T cells (Fig. 2e, f). These data indicate that IL2RG-mediated signaling is intact in the patient’s γδ T cells, while selectively impaired in his ⍺β T cells.

### Patient’s γδ T Cells Display Elevated Level of Memory Vδ2 + T Cells and Expresses Normal Level of IFN-γ Upon Stimulation

**Fig. 3:**
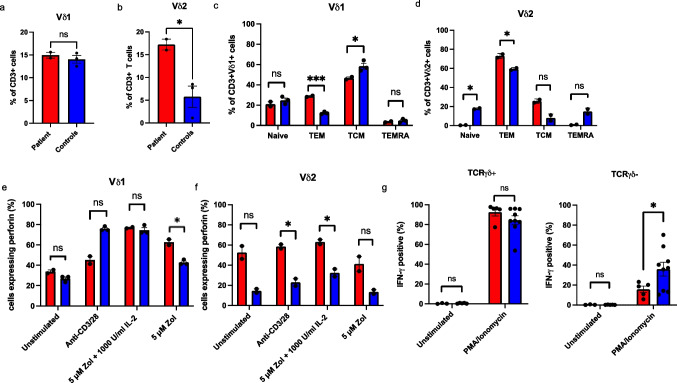
Phenotyping of the patient’s γδ T cells shows increased proportions of Vδ2 cells with high perforin expression and normal IFN-γ expression. Percentages of CD3 + lymphocytes expressing Vδ1 (**a**) and Vδ2 (**b**). Proportions of memory vs. naïve phenotypes in CD3 + Vδ1 + (**c**) and CD3 + Vδ2 (**d**) populations. Perforin expression in CD3 + Vδ1 + (**e**) and CD3 + Vδ2 + (**f**) populations in unstimulated and stimulated (1 mg/ml of anti-CD3 and 1 mg/ml anti-CD28, 5 mM of Zoledronic acid and 1000 IU/ml IL-2 or 5 mM of Zoledronic acid) conditions. **g** Intracellular IFN-γ expression in response to stimulation with PMA/ionomycin. CD3 + TCRγδ-positive cells on left and CD3 + TCRγδ-negative cells on right. Cumulative data from two independent experiments shown in **a**–**f**. In **g**, data are technical replicates combined from three independent experiments, patient *n* = 3 for unstimulated and *n* = 5 for stimulated condition, five independent controls *n* = 5 for unstimulated and *n* = 9 for stimulated condition. Error bars indicate SEM throughout the figure. ns, non-significant; *p < 0.05; ***p < 0.001 determined by unpaired *t*-test with Welch’s correction. The figure was constructed using the following software: Microsoft Office Powerpoint (64B), GraphPad Prism (v.9.2.0), FlowJo (v.10.7.2)

To further investigate the patient’s γδ T cells, we performed flow cytometric phenotyping and found that approximately 15% of the patient’s CD3 + T cells were Vδ1 + (Fig. 3a) and approximately 17% Vδ2 + (Fig. 3b). Especially the Vδ2 + population was relatively increased compared to healthy donors (1.1–8.5%). His Vδ1 + cells showed significantly higher proportion of T effector memory (TEM) cells (29% vs. 11.2–13.6%) and lower proportion of T central memory cells (TCM) (46.5% vs. 54–64.1%) while proportions of naïve and TEM re-expressing CD45RA (TEMRA) were comparable to controls (21% vs. 21–28.4% and 3.6% vs. 3–6.4%, respectively) (Fig. 3c). Essentially all patient’s Vδ2 + cells were memory T cells: 73.1% TEM (58.3–60.4% in controls) and 25.6% TCM (5.1–11.0% in controls) (Fig. 3d). In unstimulated condition, perforin expression was in Vδ1 + cells comparable to controls’ (Fig. 3e). However, approximately 53% of the patient’s Vδ2 + cells expressed perforin (vs. ca 15% in healthy controls), indicating activated state; however, the difference between patient and controls was not statistically significant (Fig. 3f). Upon stimulation, the patient’s Vδ1 + population showed somewhat lower increase in expression of perforin in response to stimulation with anti-CD3/28 but a significantly higher increase in response to zoledronic acid (Fig. 3e). The patient’s Vδ2 + cells showed an overall trend towards higher perforin expression with a significantly increased response to anti-CD3/28 and combination of zoledronic acid and IL-2 (Fig. 3f). In Fas-ligand expression, we noted a significant increase in the patient’s Vδ1 + cells and Vδ2 + cells with anti-CD3/28 and zoledronic acid stimulation, respectively (Fig. S4a-b, Online Resource). However, when the patient’s γδ T cells were activated with anti-CD3/28, the activation markers CD25 and CD69 remained lower than in healthy controls (Fig. S4c-d, Online Resource).

With PMA/ionomycin stimulation, the vast majority of the patient’s γδ T cells (approx. 95%) expressed high IFN-γ, which was comparable to healthy donors’ γδ T cells, while significantly lower proportion of his conventional CD3 + T cells became IFN-γ positive, when compared to controls (Fig. 3g). For IL-2 and IL-15, we detected no difference between the patient and controls (data not shown). Taken together, immunophenotyping of the patient’s γδ T cells shows increased memory phenotype and implies cytotoxic properties.

### The Patient’s TCRvγ Repertoire Showed a Dominance of Certain Clones

**Fig. 4:**
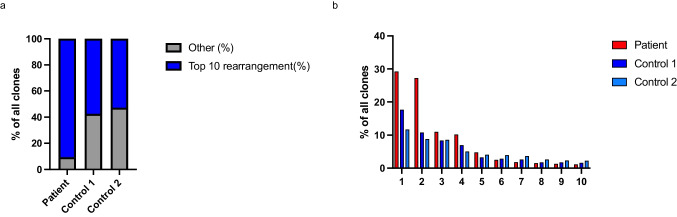
Patient’s TCRvγ repertoire is generally versatile but shows preponderance of certain clones. TCRγ sequencing results as productive frequencies of top ten clones (DNA level). Overall percentage (**a**) and distribution (**b**) of the clones in the patient’s and two healthy controls’ TCRvγ repertoire (top ten clones in blue, other clones in gray in **a**; red for the patient, blue represents control 1 and light blue control 2 in **b**). The figure was constructed using the following software: Microsoft Office Powerpoint (64B), GraphPad Prism (v.9.2.0)

To detect possible clonal expansion, we deep sequenced the patient’s TCRvγ repertoire. The top ten clones and their amino acid sequencies in the patient and healthy donors are listed in Table S4, Online Resource. On DNA level, our patient’s top ten rearrangements comprised 90.6% of all the rearrangements (controls 57.5% and 52.8%, Fig. 4a). Furthermore, the frequencies of top two clones in the patient were 29.2% and 27.2% (Fig. 4b). However, the total amount of productive rearrangements and the count of unique rearrangements were comparable to controls. These data indicate that the patient has a generally versatile TCRvγ repertoire (Table S5, Online Resource).

### Patient’s γδ T Cells Are Hyperreactive Against Malignant Cells

**Fig. 5:**
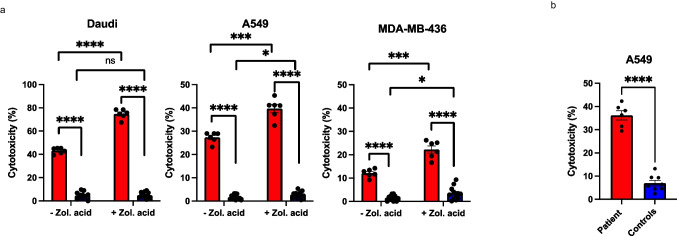
The patient’s γδ T cells exhibit enhanced and activation-independent killing ability towards malignant cell lines. **a** PBMC killing assay on Daudi cells (human Burkitt lymphoma, on the left), A549 (human adenocarcinoma, in the middle), and MDA-MB-436 (human triple breast cancer, on the right) with and without the added Zoledronic acid. Patient red, controls blue. **b** Killing of A549 by isolated and non-activated γδ T cells. Error bars represent SEM. Data are technical replicates combined from two independent experiments, for **a**: patient *n* = 6, four independent controls *n* = 12, for **b**: patient *n* = 6, four independent controls *n* = 8. ns, non-significant; *p < 0.05; **p < 0.01; ***p < 0.001; ****p < 0.0001 determined by unpaired *t*-test with Welch’s correction. The figure was constructed using the following software: Microsoft Office Powerpoint (64B), GraphPad Prism (v.9.2.0)

To determine the patient’s γδ T cells’ cytotoxic properties, we conducted killing assays using Daudi, A549, and MDA-MB-436 tumor cell lines. The patient’s PBMCs were able to induce significant lysis of all studied cell lines (Fig. 5a). Next, we repeated the killing assays with equal amounts of freshly isolated γδ T cells from the patient and healthy controls. The healthy controls showed somewhat higher lysis of the Daudi cells, but the difference was not significant (Fig.S5, Online Resource). With A549, the patient’s γδ T cells showed significantly higher cytotoxicity than controls’ γδ T cells (Fig. 5b). This data implies that the patient has a functional γδ T cell population that is hyperreactive against certain malignant cells.

### Sequencing of Patient’s *IL2RG* Revealed a Novel Somatic Variant from γδ T Cells

**Fig. 6:**
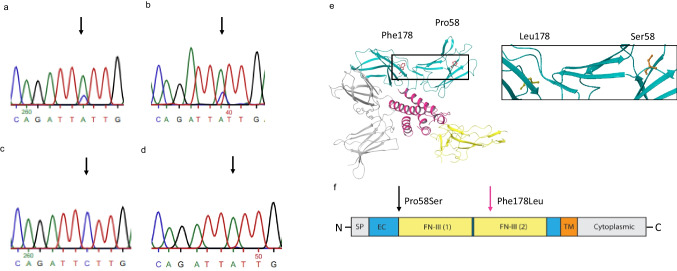
Patient’s γδ T cells contain somatic c.534C > A; p.(Phe178Leu) variant that gradually becomes predominant in the γδ cell population. Sanger sequencing of the cDNA (**a**) and gDNA (**b**) in patient’s TCRγδ + cells. **c** Sequencing of patient’s TCRαβ + cell cDNA. **d** Most recent sanger sequencing result of patient’s TCRγδ + cell gDNA. Sequencing presented here is performed on cells isolated from the patient at the age of 13 (**a**, **c**, **d**) and 12 (**b**), the time between cell isolation in **a** and **c** and in **d** was 6 months. The altered base is marked with an arrow. **e** Molecular modelling of the somatic p.(Phe178Leu) variant. **f** Schematic representation of the IL2RG domain structure. Signal peptide (SP): positions 1–22; extracellular (EC): 23–262; fibronectin type III (FN-III) (1) 59–151 and (2) 154–246; transmembrane (TM): 263–283 and cytoplasmic: 284–369 (based on NCBI Reference Sequence: NP_000197.1 and UniProtKB- P31785). Germline p.(Pro58Ser) change marked with black arrow, somatic p.(Phe178Leu) marked with pink arrow (**e**–**f**: modified form Tuovinen et al., 2020 [17]). The figure was constructed using the following software: Microsoft Office Powerpoint (64B), Adobe Illustrator, PyMOL(TM) Molecular Graphics System, Version 2.1.0. Schrodinger, LLC, Maestro Version 12.5.139, MMshare Version 5.1.139, Schrödinger Release 2020–3

To detect alterations at mRNA level, we performed RT-PCR. The only alteration when compared to reference genome was a novel c.534C > A; p.(Phe178Leu) missense variant in part of the patient’s γδ T cells (Fig. 6a). This variant was not found from the GnomAD database and was not predicted to be deleterious or pathogenic by prediction tools used (SIFT, PolyPhen2, CADD Score, Mutation Taster; Table S6, Online Resource). The same variant was also detected in the majority of the gDNA isolated from the γδ T cells (gDNA used for TCRvγ repertoire sequencing; Fig. 6b) but absent in the ⍺β T cells (Fig. 6c., Fig. S6a, Online Resource) and B lymphocytes (Fig. S6b, Online Resource), suggesting a somatic change restricted to γδ T cells. When sequencing DNA from γδ T cells after 10-day *in vitro* expansion, wild-type (WT) sequence (i.e., base C) was no longer detected (Fig. S6c, Online Resource). The sequencing of γδ T cell DNA was repeated after 1 year with similar results (Fig. 6d) indicating that the clones with this somatic variant have growth or survival advantage as WT sequence is currently absent in the γδ T cells.

### The Novel Somatic p.(Phe178Leu) Variant Affects the Stability, Protein–Protein Interaction Profile, and Plasma Membrane Targeting of IL2RG-Pro58Ser

To assess the effect of the new p.(Phe178Leu) variant on the stability of the *IL2RG* p.(Pro58Ser), we performed computational analysis with DynaMut2 tool [38]. The overall effect of Phe178Leu substitution for IL2RG was predicted to be stabilizing and IL2RG-Pro58Ser/Phe178Leu to be more stable than IL2RG-Pro58Ser variant alone (Table S7, Online Resource).

**Fig. 7:**
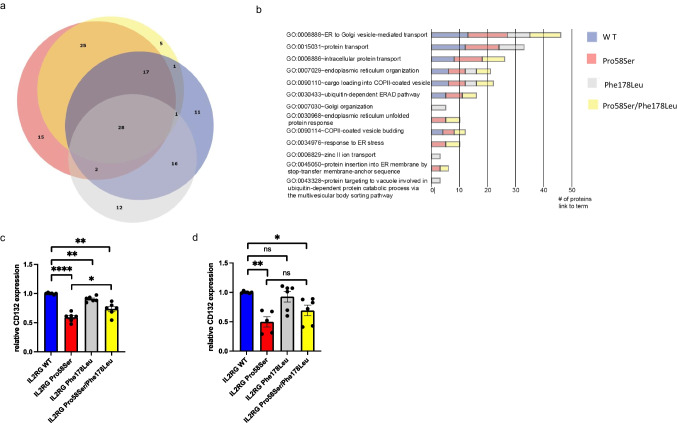
Proximal interactomes and cell surface expression of the IL2RG wild-type and the patient variants in inducible HEK293 cell lines. **a** A proportional Venn diagram illustrating the overlap and differences of the identified high-confidence interactors of the IL2RG variants. Wild-type blue, Pro58Ser red, Phe178Leu gray and Pro58Ser/Phe178Leu yellow. **b** A cluster chart displaying the number of high-confidence interacting proteins linked to enriched (*p* < 0.001) Gene Ontology terms for each of the IL2RG variants. Relative cell surface (**c**) and total cell-associated (**d**) expression of CD132 in indicated HEK293 cell lines. IL2RG WT normalized as one. In **c** and **d**, data are technical replicates combined from four independent experiments (*n* = 6 in **c** and *n* = 5–6 in **d**). Error bars indicate SEM. ns, non-significant; *p < 0.05; **p < 0.01; ****p < 0.0001 determined by unpaired *t*-test with Welch’s correction. The figure was constructed using the following software: Microsoft Office Powerpoint (64B), ProhitsViz, GraphPad Prism (v.9.2.0), FlowJo (v.10.7.2)

To study if Phe178Leu variant affects plasma membrane targeting or protein interactions of IL2RG, we performed BioID proximity labeling in inducible HEK293 cells expressing either wild-type IL2RG, IL2RG-Pro58Ser, IL2RG-Phe178Leu, or IL2RG-Pro58Ser/Phe178Leu variants [36]. According to our results, IL2RG-Pro58Ser and IL2RG-Pro58Ser/Phe178Leu displayed highly overlapping but not similar protein–protein interaction profiles whereas IL2RG-Phe178Leu had clearly more overlap with the wild-type IL2RG (Fig. 7a). However, L2RG-Pro58Ser displayed 15 significantly (≤ 0.01) increased interactions not shared by Pro58Ser/Phe178Leu or other variants. Some of these proteins were associated with endoplasmic reticulum to Golgi-mediated transport (Fig. 7a, b; Table S8, Online Resource). Furthermore, in terms of function, IL2RG-Pro58Ser/Phe178Leu had significantly lower amount of interaction with proteins associated with protein transport (GO0015031) when compared to all other IL2RG variants (*p* < 0.001, Fig. 7b). IL2RG-Pro58Ser/Phe178Leu showed significantly increased (≤ 0.01) interactions with four proteins (Table S8, Online Resource). These data suggest that the novel somatic Phe178Leu variant affects the protein–protein interaction profile of IL2RG with potential implications in cellular processes such as signaling or localization.

To assess the effect of Phe178Leu on plasma membrane targeting and protein stability, we studied the expression levels of each *IL2RG* variant in the abovementioned cell lines by flow cytometry. In line with the patient’s ⍺β cells, Pro58Ser variant showed significantly reduced cell surface expression when compared to wild-type IL2RG expressing cell line. However, the surface expression level of IL2RG-Pro58Ser/Phe178Leu variant was significantly increased when compared to Pro58Ser but remained lower than IL2RG wild-type (Fig. 7c). As expected, the total cell-associated amount of IL2RG-Pro58Ser was also significantly lower than in wild-type IL2RG cell line. The Pro58Ser/Phe178Leu variant showed a trend towards higher total protein expression level compared to Pro58Ser variant, but this did not reach statistical significance (Fig. 7d). This data suggests that somatic Phe178Leu variant partly rescues the maturation defect caused by Pro58Ser variant in stable HEK293 cell lines.

## Discussion

Expansion of γδ T cells is a previously recognized phenomenon among patients with atypical SCID [20, 22, 39]. However, the studies on function and phenotype of such atypical SCID-related γδ T cell expansion are very limited. Some reports have focused on the TCRvγ usage or γδ T cell reactivity towards CMV [18, 19, 39]. One article describes phenotypic characteristics of γδ T cells in atypical CD3δ deficiency [22], but more detailed immunophenotyping and cytotoxic properties have not been described previously.

We previously reported two patients with this novel hypomorphic *IL2RG* c.172C > T;p.(Pro58Ser) variant [17]. The second patient has been lost to follow-up without knowledge of his current γδ T cell count, which was normal at the age of 36 days. Since no evident cause for our index patient’s abnormally large γδ T cell population was found, we wanted to determine whether these γδ T cells were functional or merely a reactive expansion, possibly in response to low counts of αβ T cells. Unexpectedly, his γδ T cells showed normal IL2RG cell surface expression (Fig. 1d), cytokine signaling (Fig. 2a, b), blast formation (Fig. 2c, e), and cytotoxic abilities even superior to controls (Fig. 5), despite harboring the germline *IL2RG* p.(Pro58Ser) variant (Fig. S1b, Online Resource).

In healthy individuals, circulating γδ T cells with higher IFN-γ production expand better and display higher proportions of TCM and TEM subtypes [40]. When compared to controls, our patient’s γδ T cells displayed preponderance of TEM in both Vδ1 and Vδ2 populations and TCM in Vδ2 population (Fig. 3c, d). Of note, the patient’s Vδ2 population seems cytotoxically potent as it presents with overall tendency towards increased perforin expression. Furthermore, IFN-γ production in response to PMA-ionomycin in his γδ T cells was comparable controls (Fig. 3g). High IFN-γ secretion in general might reflect readiness to expand and may potentiate antiviral activities of γδ T cells [41–44]. Interestingly, the patient’s γδ T cells showed lower expression of activation markers CD25 and CD69 upon anti-CD3/28 stimulation (Fig. S4c-d). Activation mechanisms for these cells might thus be at least partially non-conventional.

The sequencing of the patient’s TCRγ chain revealed an accumulation of certain clones; however, no true mono- or oligoclonality was detected. Among healthy donors, certain Vγ9Vδ2 + clonotypes seem to predominate in TCR deep sequencing. Up to 80% of Vγ9 repertoire is composed of these public clonotypes putatively caused by convergent recombination [25]. In a minority of healthy donors, the top clone can comprise 20–40% of all Vγ9 and Vδ2 CDR3s. Interestingly, the most prevalent public clonotype (CALWEVQELGKKIKVF) described in many previous studies [25, 45, 46] was discovered in both controls at productive frequency of 6% but only 0.2% in the patient. Of note, while the patient’s most prevalent clone is a known Vγ9 clonotype [47], none of the five most common public Vγ9 clones listed in [25] was found in his top ten Vγ9 repertoire (Table S4, Online Resource). This data indicates a versatile and unique TCRγ repertoire, which likely did not arise merely as a reactive expansion in response to low αβ T cell percentage.

γδ T cells have been described to have oncolytic properties and have been used as cell platforms for cancer therapy [48, 49]. As the patient had high levels of γδ T cells in his peripheral blood, we wanted to test if they possessed cytotoxic abilities against tumor cell lines. With killing assays conducted on PBMCs, significantly higher degree of cytotoxicity, further increased by addition of zoledronic acid which induces expression of isopentenyl pyrophosphate (IPP), a known target for γδ T cells [50, 51], was observed (Fig. 5a). To exclude the effect of higher proportion of γδ T cells in the patient’s PBMCs, the assay was repeated with freshly isolated γδ T cells. The results were similar but interestingly, with a less sensitive cell line A549, the patient’s γδ T cells showed significantly higher cytotoxicity compared to controls’ γδ T cells (Fig. 5b) indicating a highly reactive γδ T cell pool. However, this result might possibly reflect powerful anti-tumor properties of certain clones.

While expansion of γδ T cells among patients with atypical SCID has been reported, we found only one available larger cohort study on this. In a cohort of 76 atypical SCID patients, up to 60% of patients had elevated γδ T lymphocyte proportions in their peripheral blood. This γδ T cell expansion positively correlated with CMV infection and autoimmune cytopenia, suggesting that CMV infection may drive γδ T cell expansion and that such expansions might further be causing autoimmune cytopenia [20]. *RAG1*-associated SCID and atypical SCID with CMV-driven γδ T cell expansions have been reported [18, 19], and interestingly all patients with documented CMV infection in cohort of Tometten et al. had *RAG* deficiency [20]. Since not all atypical SCID patients with γδ T cell expansions harbor CMV infection, other yet undiscovered factors must contribute to such expansions and remain to be elucidated. There is no clear indication of CMV as the driver for γδ T cell expansion in our patient; when increased γδ T cell numbers were noted, the PCR test for CMV was negative.

In their cohort, Tometten et al. found elevated γδ T cell proportions among patients with all types of atypical SCID and the authors postulated these to be a general feature of atypical SCID and not a reflection for example of αβ T cell developmental defect. However, a trend towards DNA recombinase deficient patients displaying higher amounts of γδ T cells was noted [20]. One could also speculate that partial *RAG1/2* deficiency could favor development of γδ T cells over αβ T cells, since commitment of double-negative (DN) T cells to γδ lineage takes place in DN2 stage before *RAG1/2* expression is turned on in DN3 stage allowing efficient TCRβ rearrangement and further differentiation towards αβ T cells [52].

The surface expression of IL2RG was normal on our patient’s γδ T cells, while significantly reduced on his αβ T cells (Fig. 1d, e). As the levels of total cell-associated IL2RG were decreased in both αβ T and γδ T cells (Fig. S2, Online Resource), increased transcription is unlikely the mechanism behind normal cell surface expression in γδ T cells. We found a novel *IL2RG* c.534C > A; p.(Phe178Leu) variant in our patient’s γδ T cells (Fig. 6). This somatic variant was the only genomic alteration in addition to c.172C > T;p.(Pro58Ser) discovered in his γδ or αβ T cells and was not detected in the original exome sequencing performed at the age of 8. Somatic variants in lymphocytes are common in healthy individuals and patients with inborn errors of immunity [53]. However, they usually are restricted to a single or certain clones rather than whole lymphocyte subset (variant allele frequency is relatively low). As initially only part of the patient’s γδ T cells carried the p.(Phe178Leu) variant (Fig. 6) and the expansion was originally not monoclonal (Fig. 4a, b), the DNA change has occurred in a progenitor cell level either in bone marrow or thymus. Hypothetically, there could be a common precursor that produces exclusively γδ T cells, or double-negative (DN) T cells with IL2RG p.(Phe178Leu) variant may carry selective advantage to differentiate to TCRγδ +rather than TCRαβ + T cells in thymus. However, this most likely is not caused by increased IL-7R-mediated signaling as the patient’s TCRγδ+ cells did not display enhanced responsiveness to IL-7 (Fig. S3c, Online Resource). The majority of the patient’s γδ T cells were Vγ9Vδ2+ , a population known to be influenced by both pre- and postnatal events [25]. In conclusion, it can be hypothesized that at some point in the patient’s γδ T cell ontogeny p.(Phe178Leu) has conferred advantage in function and/or survival. This hypothesis is further strengthened by the observed enrichment of the γδ T cell population harboring IL2RG p.(Phe178Leu) variant after *in vitro* expansion and over time in the patient (Fig. S6c, d) and with predicted stabilizing effect of the p.(Phe178Leu) variant on IL2RG p.(Pro58Ser) (Table S7, Online Resource). The protein interaction profile of IL2RG-Pro58Ser/Phe178Leu variant was found to be slightly different to IL2RG-Pro58Ser or wild-type IL2RG (Fig. 7a, b). As shown before, IL2RG-Pro58Ser variant showed increased interactions with ER/Golgi and nuclear proteins (Fig. 7b) [17]. Our BioID analysis revealed 15 significantly increased protein interactions limited to IL2RG-Pro58Ser variant (Table S8, Online Resource). As these interactions — including the abovementioned ER/Golgi proteins — were not shared with the IL2RG-Pro58Ser/Phe178Leu, it is possible that they are contributing to the observed difference in plasma membrane targeting. Furthermore, IL2RG-Pro58Ser/Phe178Leu variant showed enhanced plasma membrane targeting compared to Pro58Ser variant alone in stable HEK293 cell lines (Fig. 7c). In silico, Phe178Leu is predicted to be stabilizing (Table S7, Online Resource) and possibly due to this stabilizing effect the total cell-associated levels of IL2RG-Pro58Ser/Phe178Leu are reduced to a lesser degree than IL2RG-Pro58Ser when compared to IL2RG WT in our HEK293 cell lines (Fig. 7d) as well as in the patient’s γδ T versus his ⍺β T cells (Fig. 1d, e).

However, the physiological meaning of these findings cannot be directly translated to *in vivo* conditions as numerous factors might contribute to plasma membrane targeting in cell type–specific manner. Furthermore, Phe178Leu change can also affect interactions with other receptor subunits associated with IL2RG signaling expressed in lymphocytes but not in HEK293 cells. These differences between cell types could explain why IL2RG-Pro58Ser/Phe178Leu cell surface levels are not equal to wild-typein our HEK293 cell model. However, since the patient’s γδ T cells with the somatic variant display normal cell surface expression levels CD132 and as the IL2RG-Pro58Ser/Phe178Leu variant displays higher cell surface levels than IL2RG-Pro58Ser in HEK293 cell lines, it can be concluded that Phe178Leu is at least partially rescuing the effect of Pro58Ser in terms of plasma membrane targeting. As Phe178Leu variant is in the extracellular domain of IL2RG, it could theoretically alter its cytokine binding abilities. However, as these γδ T cells are hyperreactive towards certain cancer cell lines (Fig. 5b), there might be other undiscovered factors explaining certain functional properties.

Since the IL2RG p.(Phe178Leu) second-site genetic alteration appears to — at least partially — abrogate the negative effect of the germline Pro58Ser variant in terms of localization, it can be considered as a second-site genetic reversion. To our knowledge, this is the first in-depth report of characterizing the functionality of expanded γδ T cell population in an individual with atypical SCID and describing positively affecting somatic second-site reversion restricted to γδ T cells. We sought for more atypical SCID patients with no success, probably due to the rareness of the disease. However, based on our findings, further investigation of the functional abilities of expanded γδ T cell populations in patients with atypical SCID will be needed.

## Supplementary Information

Below is the link to the electronic supplementary material.

Supplementary file1 (DOCX 627 KB)

Supplementary file2 (XLSX 58 KB)

## Data Availability

The datasets generated during and/or analyzed during the current study are available from the corresponding author on reasonable request.
