# Supplementary material for: Characterization of Expanded Gamma Delta T Cells from Atypical X-SCID Patient Reveals Preserved Function and IL2RG-Mediated Signaling
Source: J Clin Immunol. 2022 Oct 19;43(2):358–70. doi: 10.1007/s10875-022-01375-6 (PMC9892142; doi:10.1007/s10875-022-01375-6)
Supplement: Supplementary file 1 — Supplementary file1 (DOCX 627 KB) [file 10875_2022_1375_MOESM1_ESM.docx]

**Online Resource** **(supplementary text, tables and figures)**

**Characterization of expanded gamma delta T cells from atypical X-SCID patient reveals preserved function and IL2RG-mediated signaling**

**in Journal of Clinical Immunology**

Elina A. Tuovinen^1,2,3^, Sakari Pöysti^4^*, Firas Hamdan^1,5,6^*, Kim My Le^1,3^, Salla Keskitalo^7^, Tanja Turunen^7^, Léa Minier^1,8^, Nanni Mamia^1^, Kaarina Heiskanen^3,9^, Markku Varjosalo^7^, Vincenzo Cerullo^1,5,6^, Juha Kere^2,10,11^, Mikko R.J. Seppänen^1,3,12^, Arno Hänninen^4^, Juha Grönholm^1,3^†

^1^ Translational Immunology Research Program, University of Helsinki, Helsinki, Finland; ^2^ Folkhälsan Research Center, Helsinki, Finland; ^3^ Pediatric Research Center, New Children’s Hospital, University of Helsinki and HUS Helsinki University Hospital, Helsinki, Finland; ^4^ Department of Clinical Microbiology and Immunology, Turku University Hospital, Turku, Finland; ^5^ Drug Research Program Helsinki (DRP), Faculty of Pharmacy, University of Helsinki, Helsinki, Finland; ^6^ Digital Precision Cancer Medicine Flagship (iCAN), University of Helsinki, Helsinki, Finland; ^7^ Systems Biology Research Group and Proteomics Unit, Institute of Biotechnology, HiLIFE, University of Helsinki, Helsinki, Finland; ^8^ Faculty of Science and Technology, University of Lille, Lille, France ^9^ Children´s Immunodeficiency Unit, New Children’s Hospital, University of Helsinki and HUS Helsinki University Hospital, Helsinki, Finland; ^10^ Department of Biosciences and Nutrition, Karolinska Institutet, Stockholm, Sweden; ^11^ Stem Cells and Metabolism Research Program, University of Helsinki, Helsinki, Finland; ^12^ Rare Diseases Center and Pediatric Research Center, New Children’s Hospital, University of Helsinki and HUS Helsinki University Hospital, Helsinki, Finland

* Equal contribution

† Corresponding author

Corresponding author: Juha Grönholm; email: [juha.gronholm@helsinki.fi](mailto:juha.gronholm@helsinki.fi)

**Clinical update of the index patient**

The index is a 13-year-old male born to nonconsanguineous Finnish parents after an uneventful pregnancy. He has suffered from recurrent respiratory infections, reactive arthritis and bronchiectasis. His detailed and extended case report has been described earlier [1]. Since the age of nine, the bronchiectasis has shown no progression in HRCT imaging. The patient is currently still treated with IVIG and prophylactic azithromycin and has had no respiratory infections. He has suffered from secondary bacterial infections associated to molluscum contagiosum that have been treated with cephalexin on two occasions at the age of 12. Suspicion has been raised whether the patient is malnourished, and he has been referred to nutrition therapist. He displayed growth spurt at the age of 12. Currently his growth is at -1SD and he is staged G3-4P3-4 on Tanner scale. Except constipation treated with polyethylene glycol, he hasn’t suffered from any other gastrointestinal symptoms or evident signs of malabsorption. CMV nucleic acid was tested negative at the age of seven but at the age of eleven CMV IgG was positive and IgM negative possibly indicating old immunity. On the other hand, this could be due to IVIG started at the age of nine. Of note, the patient has not displayed any clinical signs of CMV infection.

**Cell isolation**

Blood samples were drawn into lithium heparin vacuum tubes, and until further processed kept at room temperature. Blood samples were drawn simultaneously from index patient and two or three healthy controls (aged 10-50) Peripheral mononuclear cells (PBMCs) were isolated using Ficoll Paque-gradient centrifugation (Ficoll-Paque, Pharmacia and Ficoll-Paque plus, GE Healthcare). Freshly isolate PBMCs were subjected to Magnetic activated cell sorting (MACS) with TCRγ/δ^+^T Cell Isolation Kit (Miltenyi Biotec) according to manufacturer’s instructions. Cell isolation of other lymphocyte subpopulations is described elsewhere [1].

**Blasting, expansion, sorting and isolation of TCRαβ+ and TCRγδ+ cells**

The protocol for TCRγδ+ cell expansion has been described elsewhere [2]. Briefly, freshly isolated PBMCs were resuspended RPMI 1640 medium (Gibco, Thermo Fischer Scientific) supplemented with 10% FBS (HyClone), 2mM L-Glutamine and 100IU/ml penicillin-streptomycin (Gibco, Thermo Fischer Scientific) at a concentration of 10^6 cells/ml. The cells were initially stimulated with 5μM zoledronic acid (Merck) and 1000 IU/ml IL-2 (R&D systems). Medium containing 1000 IU/ml IL-2 was added every 2-3 days. On day 10, TCRγδ+ cells were isolated with either negative selection (MACS) as described above or by FACS alongside with TCRαβ+ cells. For FACS, the cells were stained with fluorescent-conjugated antibodies for CD3 (BD Biosciences), TCRαβ (BioLegend) and TCRγδ (BD Biosciences) and further sorted using BD Influx sorter. In the case with no prior blasting/expansion, TCRγδ and TCR⍺β T cells were FACS sorted from freshly isolated PBMC:s using BD Influx sorter as previously described.

**TCRγ receptor sequencing**

10^6 freshly MACS separated TCRγδ+ cells were used as starting material. Sample data was generated using the immunoSEQ® Assay (Adaptive Biotechnologies, Seattle, WA) at deep sequencing level. The somatically rearranged TCRγ CDR3 was amplified from genomic DNA using a two-step, amplification bias-controlled multiplex PCR approach [3,4]. The set of observed biological TCRγ CDR3 sequences were normalized to correct for residual multiplex PCR amplification bias and quantified against a set of synthetic TCRγ CDR3 sequence analogues. Data was analyzed using the immunoSEQ Analyzer toolset.

**Flow cytometry**

Expression of cell surface IL2RG (CD132) was analyzed from TCR γδ cells and TCR γδ negative CD3+ T cells from whole blood. 100µl of whole blood was incubated with fluorescent-conjugated antibodies for CD3 (eBioscience), CD132 (eBioscience) and TCRγδ (BD Biosciences) on ice for 15 min. Red blood cells were lysed with BD FACS Lysing Solution and cell were analyzed with flow cytometry (NovoCyte model 3000 and NovoExpress, Acea). To evaluate proportion of γδ and ⍺β TCR cells within CD3+ T cells PBMCs were stained with fluorescent-conjugated antibodies for CD3 (BD Pharmigen), TCR γδ (eBioscience) and TCR ⍺β (BioLegend) for 30 min at +4°C. For intracellular staining of CD132, surface stained PBMCs were fixed with LyseFix-buffer (eBioscience) and permeabilized with 90% methanol in PBS over night at -20°C followed by staining with anti-CD132 (BioLegend) and analysis with BD Fortessa flow cytometer and FlowJo (v10.7.2) software.

**STAT phosphorylation in response to exogenous IL-2, IL-4, IL-7, IL-15 and IL-21**

To measure STAT5 phosphorylation, isolated PBMCs were stimulated with IL-2 (10 U/ml and 320 U/ml, R&D Systems) or IL-15 (6ng/ml and 100ng/ml, R&D Systems) for 15 min in pre-warmed (37°C) RPMI 1640 medium (Gibco, Thermo Fischer Scientific) supplemented with 10% FBS (HyClone), 2mM L-Glutamine and 100IU/ml penicillin-streptomycin (Gibco, Thermo Fischer Scientific). Stimulated cells were fixed with Cytofix fixation buffer (Becton Dickinson) and permeabilized with Phosflow Perm Buffer IV (BD) according to manufacturer’s protocol. Cells were stained with fluorescent-conjugated antibodies for CD3 (eBioscience), TCR γδ (BD Bioscience) and pY-STAT5 (eBioscience). Stained cells were analyzed by flow cytometry (NovoCyte model 3000/BD and NovoExpress software, Acea). To measure STAT3, ~~and~~ STAT6 and STAT5 tyrosine phosphorylation, PBMCs were stimulated with 50 ng/ml IL-21 (Peprotech), ~~or~~ ~~with~~ 100 ng/ml IL-4 (Biotechne)~~,~~ or 50ng/ml IL-7 (Miltenyi Biotec) respectively, together with surface staining with fluorescent-conjugated antibodies for CD3 (BD Pharmigen), TCR γδ (eBioscience) and TCR ⍺β (BioLegend) for 15 min in +37°C water bath. Cells were fixed by adding 1ml prewarmed lysefix buffer (BDBiosciences) and incubated with additional 12 min at +37°C. Cells were permeabilized with 90% methanol in PBS over night at -20°C followed by staining with anti-STAT3 pY705, anti-STAT3 pY705 , anti-STAT6 pY641 or anti-STAT5 pY694 (all from BD Biosciences). Cells were further analyzed using BD Fortessa flow cytometer and FlowJo (v10.7.2) software.

**Flow cytometric assay for specific cell-mediated immune-responses in activated whole blood (FASCIA)**

Blast formation of TCR γδ cells was determined by FASCIA described in [5]. Briefly, whole blood from heparinized tubes was diluted in RPMI (containing Glutamax I, gentamicin and β-mercaptoethanol) and stimulated with IL-2 (10 U/ml and 320 U/ml, R&D Systems) or IL-15 (6ng/ml and 100ng/ml, R&D Systems) for 5 days. Stimulated cells were then stained with fluorescent-conjugated antibodies for CD45, CD3 (both from eBioscience) and TCR γδ (BD Biosciences). Red blood cells were lysed with FACS lysing solution (BD) and blast transformation of gated TCR γδ cells was analyzed by their light scatter characteristics using flow cytometry (NovoCyte model 3000 and NovoExpress software, Acea).

**Analysis of CD25 and CD69 cell surface expression and intracellular cytokines**

For analysis the CD25 and CD69 upregulation in response to TCR stimulation, 200 000 PBMCs/well were plated on a 96-well plate in RPMI 1640 medium (Gibco, Thermo Fischer Scientific) supplemented with 10% FBS (HyClone), 2mM L-Glutamine and 100IU/ml penicillin-streptomycin (Gibco, Thermo Fischer Scientific). The cells were incubated for 24h with or without plate bound antibodies for anti-CD3/28 (BioLegend). Cells were then fixed with 4% PFA and stained with fluorescent-conjugated antibodies for CD3, TCR γδ, CD25 (all from BD Biosciences) and CD69 (BioLegend). For analysis of intracellular cytokine expression, 10^6^ PBMCs were serum starved in RPMI medium W/O added FBS (described above) at +37C for 2 hours. PBMCs were then stimulated with Biolegend's Activation cocktail (Phorphol 12-myristate 13-acetate (PMA), ionomycin and Brefeldin A for 6 hours at +37C, followed by staining with fixable viability dye according to manufacturer's instructions (eFluor506 fixable viability dye, eBioscience). Cells were then fixed and permeabilized according to manufacturer’s protocol (BD Biosciences) and stained with fluorescent-conjugated antibodies for CD3, TCR γδ (both from BD Biosciences), CD4, CD8, IL-2, IL-4 (all from BioLegend), IL-15 (Invitrogen) and IFN-γ (BD Biosciences). Cells were analyzed using Fortessa flow cytometer and FloJo (v10.7) software.

**LDH release assay**

Killing assays were performed by plating 15,000 cells in a 96 well plate. After plating, PBMCs or isolated γδT cells were added at a 1:40 or 1:20 (Target:Effector) ratio, respectively. Co-cultures were incubated for 4 hours at 37°C and supernatants were then collected. LDH releases was then calculate using the CyQUANT LDH Cytotoxicity assay kit (Invitrogen) and following manufacturer’s instructions. Specific lysis was then calculated using the following formula:

$\frac{Experimental LDH-(Effector LDH+Target LDH)}{Maximum LDH- (Effector LDH+Target LDH)}$ X100

Experimental LDH corresponds to when both effector and target cells are incubated, Effector LDH corresponds to when only effector cells are present, Target LDH corresponds to only when target cells are plated and Maximum LDH is when target cells were treated with cell lysis buffer. Cells used as target cells were A549 (human adenocarcinoma), MDA-MB-436 (human triple breast cancer) and Daudi cells (human Burkitt lymphoma). A549 cells were cultured in low glucose DMEM (Gibco) while for MDA-MB.436 cells were cultured in high glucose DMEM (Gibco). Daudi cells, PBMCs and isolated gamma delta T cells were all grown in low glucose RPMI (Gibco). All mediums were supplemented with 10% FBS, 100 IU/ml penicillin/streptomycin and L-glutamine.

***In silico* computational analysis**

The structure of the cytokine receptor complex (PDB: 2B5I) was obtained from RCSB Protein Data Bank (https://www.rcsb.org/) and visualized with Maestro software (Maestro Version 12.5 .139, Release 2020-3). The mutations to the IL2RG chain were created with the mutagenesis tool in PyMol software (PyMOL™ Molecular Graphics System, Version 2.1.0.) by using PDB: 2B5I as a template. DynaMut2 [6] was used to carry out stability predictions of the mutated protein structures.

**Generation of IL2RG expression constructs**

The new somatic Phe178Leu variant was introduced to C-terminal Mac-Tag gateway destination vectors containing either *IL2RG* WT sequence or Pro58Ser variant using QuikChange II Site-Directed Mutagenesis Kit (Agilent Technologies) according to manufacturer’s instructions. Primers used in the plasmid mutagenesis are listed in the Table S1.

**Flp-In 293 Trex cell line generation**

Stable cell lines were generated from HEK293 Flp-In Trex cells with different *IL2RG* constructs (described above) with C-terminal MAC tags [7]. As described previously [8], the cell lines were grown to 80% confluency and induced with 2 μg/ml tetracycline and 50 μM biotin for 24 hours, harvested and lysed and affinity-purified according to the BioID sample preparation workflow.

**Sample preparation and proteomic analysis**

After reduction and alkylation, samples were trypsin digested overnight and desalted with C18 BioPureSPN MINI columns (Nest Group, Ispwich, MA, USA) according to manufacturer’s instructions. The dried peptides were reconstituted in 30 µl Buffer A (0.1% (vol/vol) TFA and 1% (vol/vol) acetonitrile (ACN) in HPLC water). Samples were further diluted 1+19 µl with HPLC water containing 0.1 vol/vol% formic acid. The manufacturer's instructions were followed to load into Evotips (Evosep, Denmark). The desalted samples were analyzed using the Evosep One liquid chromatography system coupled to a hybrid trapped ion mobility quadrupole TOF mass spectrometer (Bruker timsTOF Pro 2) via a CaptiveSpray nano-electrospray ion source (Bruker Daltonik GmbH, Germany). An 8 cm × 150 µm column with 1.5 µm C18 beads (EV1109, Evosep) was used for peptide separation with the 60 samples per day methods (21 min gradient time). Mobile phases A and B were 0.1 % formic acid in water and 0.1 % formic acid in acetonitrile, respectively. The MS analysis was performed in the positive-ion mode using data-dependent acquisition (DDA) in PASEF [9] mode with DDA-PASEF-short_gradient_0.5s-cycletime -method.

**Mass spectrometry data processing**

Raw data (.d) were processed with FragPipe v18 utilizing MSFragger [10–12] against the reviewed Human proteome UP000005640. Decoy sequences and common contaminants were generated and added to the original database as part of the FragPipe workflow. Carbamidomethylation of cysteine residues was used as static modification. Amino terminal acetylation and oxidation of methionine were used as the dynamic modification. Biotinylation of lysine and N-termini were set as variable modifications. Trypsin was selected as enzyme, and maximum of two missed cleavages were allowed. The allowed peptide length and mass ranges were 5–50 residues and 200–5000 Da, respectively. Within FragPipe all peptide-spectrum matches (PSMs), peptides, and proteins were filtered to 1% PSM and 1% protein FDR. For MSFragger precursor tolerance was set to 50 ppm and fragment tolerance was set to 20 ppm, with mass calibration and parameter optimization enabled. Isotope error was set to 0/1/2. The minimum number of fragment peaks required to include a PSM in modeling was set to two, and the minimum number required to report the match was four. The top 150 most intense peaks and a minimum of 15 fragment peaks required to search a spectrum were used according to recommended settings. Label-free quantification was employed with default settings.

The resulting protein detections were filtered using Significance Analysis of INTeractome [13], Contaminant Repository for Affinity Purification [14] and MAC-tagged GFP control interactions. Interactors with < 0.01/0.05 BFDR and Crapome ≤ 20 % match were chosen as the filtered high-confidence interactors. Results were visualized with ProhitsViz.

**CD132 expression in Flp-In 293 Trex cells**

Previously described stable HEK293 Flp-In Trex cell lines were used to assess total cell associated and cell surface expression levels. Approximately 600 000 cells/well were plated on 6-well plate and incubated at 37°C in 2ml DMEM medium (Gibco, Thermo Fischer Scientific) supplemented with 10% FBS (HyClone), 2mM L-Glutamine and 100IU/ml penicillin-streptomycin (Gibco, Thermo Fischer Scientific). Next day, medium was replaced with fresh medium containing 2 μg/ml tetracycline. After 24h incubation, the cells were detached from the wells with Trypsin-Versene (Lonza). The cells were then either fixed with 4% PFA in PBS or fixed and permeabilized with BD Cytofix/Cytoperm (BD BioSciences). The cells were stained with fluorescent-conjugated antibody for CD132 (eBioscience, Biolegend) and further analyzed using BD Fortessa flow cytometer and FlowJo (v10.7.2) software.

**Table S1** Primers used in RT-PCR, Sanger sequencing and plasmid mutagenesis

| Exon 1 cDNA FWD | ACC CAG GGA ATG AAG AGC AAG |
| --- | --- |
| Exon 1 cDNA REV | GGC GTC AGA ATT GTC GTG TT |
| Exon 1 cDNA FWD | AGA CAG ACT ACA CCC AGG GAA |
| Exon 1 cDNA REV | TGG GCG TCA GAA TTG TCG TG |
| Exon 1-5 cDNA FWD | AGA CAG ACT ACA CCC AGG GAA |
| Exon 1-5 cDNA REV | TGG GGT GGC TCC ATT CAC TC |
| Exon 2-5 cDNA FWD | GCC TAC CAA CCT CAC TCT |
| Exon 2-5 cDNA REV | CCG AAC ACG AAA CGT GTA |
| Exon 5-8 cDNA FWD | CCT TGC CTA GTG TGG ATG GG |
| Exon 5-8 cDNA REV | GGG GTT CAG GTT TCA GGC TT |
| Exon 4-6 cDNA FWD | ACA GGC CAC ACA GAT GCT AAA |
| Exon 4-6 cDNA REV | TAT TGC TCC CCC AGT GGA TTG |
| Exon 8 cDNA FWD | TCT GTT GGC TCC ATG GGA TT |
| Exon 8 cDNA REV | GAA GGG GTG TTA CAT GGG G |
| Exon 8 cDNA FWD | TCA TTC AAC CCA CCT GCG TC |
| Exon 8 cDNA REV | AAC TTT ATT TCT CAT CGG TTC AGG A |
| Exon 4 Phe178Leu gDNA FWD | GGG CTC CAG AGA ACC TAA CAC |
| Exon 4 Phe178Leu gDNA REV | ACG TCC CTA GTC ACT CAC AGT |
| IL2RG Pro58Ser gDNA FWD | TGG CTG CAC TTC TGG ACT TT |
| IL2RG Pro58Ser gDNA REV | CCC TCC CTG CCT TCA TTT TCT |
| IL2RG Phe178Leu FWD (mutagenesis) | CTG AAC TGG AAC AAC AGA TTA TTG AAC CAC TGT TTG GAG C |
| IL2RG Phe178Leu REV (mutagenesis) | GCT CCA AAC AGT GGT TCA ATA ATC TGT TGT TCC AGT TCA G |

**Table S2** Monoclonal antibodies used

| **Antibody** | **Fluorochrome** | **Clone** | **Manufacturer** |
| --- | --- | --- | --- |
| TCR γδ | FITC | B1 | BD Biosciences |
| TCR γδ | FITC | B1.1 | eBioscience/Invitrogen |
| TCR γδ | PE-CF594 | B1.1 | BD Biosciences |
| TCR ⍺β | BV421 | IP26 | BioLegend |
| CD3 | PE/Cy7 | UCTH1 | BioLegend |
| CD3 | Pacific Blue | UCTH1 | BioLegend |
| CD3 | eFluor506 | UCHT1 | eBioscience |
| CD3 | AF700 | UCHT1 | BD Pharmigen |
| CD3 | - | HIT3a | BioLegend |
| CD28 | - | B28.2 | BioLegend |
| CD132 | PE | TUGh4 | eBioscience |
| CD132 | APC | TUGh4 | BioLegend |
| CD132 | - | A10 | Santa Cruz |
| CD4 | BV421 | IP26 | BioLegend |
| CD4 | PE/Cy5 | OKT4 | Biolegend |
| CD8 | APC-Cy7 | RPA-T8 | BioLegend |
| CD8 | PerCP | HIT8A | Biolegend |
| CD14 | FITC | 63D3 | BioLegend |
| CD14 | V500 | MΦPg | BD Horizon |
| CD45 | APC-eFluor780 | HI30 | eBioscience |
| CD19 | BV605 | HIB19 | BioLegend |
| CD25 | APC-H7 | M-A251 | BD Biosciences |
| CD25 | PE | BC96 | BioLegend |
| CD27 | PerCP | O323 | BioLegend |
| CD56 | FITC | MEM-188 | Biolegend |
| CD62L | APC | DREG-56 | BioLegend |
| CD69 | FITC | FN50 | BioLegend |
| CD69 | APC/Cyanine 7 | FN50 | Biolegend |
| STAT3 pY705 | PE | 4 | BD Biosciences |
| STAT3 | PerCP-Cy5.5 | M59-50 | BD Biosciences |
| pSTAT5 pY694 | AF647 | 47/Stat5(pY649) | BD Biosciences |
| pSTAT5 pY694 | PE | SRBCZX | eBioscience |
| pSTAT6 pY641 | PE | 18 | BD Biosciences |
| IFNγ | BV711 | B27 | BD Biosciences |
| HA | - | 16B12 | BioLegend |
| IL-2 | FITC | MQ1-17H12 | BioLegend |
| IL-15 | APC | 34559 | Invitrogen |
| IL-4 | PE-Cy7 | MP4-25D2 | BioLegend |
| Vdelta 1 | PE | REA173 | Miltenyi Biotec |
| Vdelta 2 | AF700 | B6 | Biolegend |
| Perforin | Pacific Blue | dG9 | Biolegend |
| Granzyme B | AF647 | QA16A02 | Biolegend |
| CD107a | APC | H4A3 | Biolegend |
| CD178 | BV421 | NOK-1 | BD Bioscience |

**Table S3** Immunologic characteristics of the index patient

|  |  | **Healthy control median/ reference range (cells/μl)** | **Patient**  **10 yo** | **12 yo** | **13 yo** |
| --- | --- | --- | --- | --- | --- |
| **Leukocytes** |  | **4500-13500/ μl** | **7200/ μl** | **n/a** | **5300/μl** |
|  | Lymphocytes | 13-48% | 42.6%; 3070/ μl | n/a | 2950/ μ; 55%; |
|  | Monocytes | 4-9% | 12.5%; 900/ μl | n/a | 480/μl;9% |
|  | Neutrophils | 36-77% | 41.0%; 2950/ μl | n/a | 1680/μl; 32% |
|  | Basophils | 0-1% | 1.3%; 90/ μl | n/a | 60/μl;1% |
|  | Eosinophils | 0-6% | 2.4%; 170/ μl | n/a | 150/μl; 3% |
|  | Platelets | 200000-450000/ μl | 302 000/ μl | n/a | 269 000/μl |
| **Dendritic cells ^a^**  **(of lymphocytes)** |  |  |  | **n/a** | **n/a** |
| Plasmacytoid | lin^-^HLA-DR^+^CD123^+^CD11c^-^ | 0.1-0.3% | 0.02% |  |  |
| Monocytoid | lin^-^HLA-DR^+^CD123^-^CD11c^+^ | 0.1-0.3% | 0.13% |  |  |
| **CD3+ T cells ^a^** |  | **750-2760/ μl; 56-86% (of lymphocytes)** | **1880/** **μl; 65%** | **1320/μl; 66%** | **1930/μl; 66%** |
|  | TCRαβ+ | 88.1-97.8% | 64.1% | 62,7 % | n/a |
|  | TCRγδ+ | 1.9-11.7% | 35.9% | 37.3% | n/a |
|  | Naive CCR7+CD45RA+ | - | 40.2% | 43.7% | n/a |
|  | Memory CD45RO+ | - | 38.3% | 42.9% | n/a |
|  | CD4+CD8+ | 0.3-3.3 | 0.2% | 0.1% | n/a |
|  | CD4-CD8- | 3.1-9.3% | 32.4% | 30.9% | n/a |
|  | TCRαβ^+^ CD4-CD8- | - | 1.5% | 1.3% | n/a |
| T_reg_ | FOXP3^+^CD25^high^CD127^low^ | 2.8-6.4% | 3.3% | 3.6% | n/a |
| **Activated** | HLA-DR+ CD38- | - | 9.8% | 8.4% | n/a |
|  | HLA-DR-CD38+ | - | 46.5% | 47.5% | n/a |
|  | HLA-DR+ CD38+ | - | 10.5% | 5.0% | n/a |
| **CD4/CD8 ratio** |  | 0.8-3.7 | 0.6 | 0.7 | 0.7 |
| **CD3+CD4+ T cells** |  | **404-1612/** **μl; 33-58%** | **503/** **μl;17%** | **384/μl; 29%** | **675/μl; 24%** |
| Naive | CCR7+CD45RA+ | 20.5-54.8% | 49.7% | 49.3% | n/a |
| TCM | CCR7+CD45RA- | 8.4-32.8% | 43.1% | 43.8% | n/a |
| TEM | CCR7-CD45RA- | 19.9-52.4% | 7.0% | 6.7% | n/a |
| Temra | CCR7-CD45RA^+^ | 1.4-17.0% | 0.2% | 0.5% | n/a |
| T_reg_ | FOXP3^+^CD25^high^CD127^low^ | - | 13.4% | 13.6% | n/a |
|  | CD45RA+CD62L+ | 27.4-64.7% | 52.0% | 51.1% | n/a |
| Recent thymic emigrants (RTE) | CD45RA+CD62L+CD31+ | 14.4-38.3% | 38.9% | 41.7% | n/a |
| Activated | HLA-DR+ CD38- | 2.4-9.6% | 4.6% | 5.8% | n/a |
|  | HLA-DR-CD38+ | 40.4-72.9% | 67.1% | 70.4% | n/a |
|  | HLA-DR+ CD38+ | 0.9-4.6% | 3.6% | 3.3% | n/a |
| **CD3+CD8+ T cells** |  | **220-1130/** **μl; 13-39%** | **860/** **μl; 30%** | **590/μl;44%** | **970/ μl; 32%** |
| Naive | CCR7+CD45RA+ | 18.8-71.0% | 61.8% | 66.0% | n/a |
| TCM | CCR7+CD45RA- | 1.2-7.3% | 1.1% | 2.9% | n/a |
| TEM | CCR7-CD45RA- | 14.6-63.0% | 12.4% | 17.8% | n/a |
| Temra | CCR7-CD45RA^+^ | 4.5-33.7% | 24.7% | 13.3% | n/a |
| Activated | HLA-DR+ CD38- | 3.8-32.4% | 5.0% | 4.5% | n/a |
|  | HLA-DR-CD38+ | 30.3-78.5% | 62.1% | 48.5% | n/a |
|  | HLA-DR+ CD38+ | 1.4-21.4% | 6.0% | 2.8% | n/a |
| **CD19+ B cells ^b^** |  | **80-620/** **μl; 5-22% (of lymphocytes)** | **500/ μl; 18%** | **380/μl; 19%** | **690/ μl; 24%** |
| Naive | CD27-IgD+IgM+ | 51.0-83.0% | 88.2% | n/a | n/a |
| Memory | CD27+ | 13.0-48.0% | 9.6% | n/a | n/a |
| Marginal zone-like | CD27+IgD+IgM+ | 5.0-18.0% | 6.7% | n/a | n/a |
| Switched memory | CD27+IgD-IgM- | 9.0-26.0% | 2.1% | n/a | n/a |
| Activated | CD38^low^CD21^low^ | 3.0-9.0% | 8.2% | n/a | n/a |
| Transitional | CD38++IgM++ | 1.0-13.0% | 3.8% | n/a | n/a |
| Plasmablasts | CD38++IgM- | 1.0-7.0% | 0.3% | n/a | n/a |
| **CD3+CD16^+^/56^+^** **NK cells** |  | **80-720/ μl; 5-26%** | **480/ μl; 17%** | **280/μl; 14%** | **260/μl; 9%** |
| **Serum Immunoglobulins**  **(prior IVIG)** | IgG HUSLAB | 5.6-19.2 g/L | 7.8 g/L | n/a | n/a |
|  | IgA | 0.35-2.99 g/L | 1.92 g/L | n/a | n/a |
|  | IgM | 0.36-2.07 g/L | 2.45 g/L | n/a | n/a |
|  | IgE | 0-320 IU/L | 14 IU/L | n/a | n/a |
| **Lymphocyte proliferative responses to mitogens** | Phytohemagglutinin (PHA), Concanavalin A (ConA), Pokeweed mitogen (PWM) | | CD4+: PHA↓(slightly decreased), ConA n/↓(decreased), PWM n  CD8+: PHA n, ConA n/↓(decreased), PWM n  CD19+: PWM n/↓ (clearly decreased) | n/a | n/a |
| **Specific antibodies against vaccine antigens** | Anti-tetanus, anti-pneumococcal  Anti-diphtheria, Anti-haemophilus influenzae | | Tetanus: n; Pneumococcal (conjugate) n/ (polysaccharide) ↓;  dipht. ↓; Haemph. ↓ | n/a | n/a |

When available, in-house (HUSLAB) determined pediatric reference values were used. Other reference values: ^a^HUSLAB reference values for adults; ^b^Piątosa B, Wolska-Kuśnierz B, Pac M, Siewiera K, Gałkowska E, Bernatowska E. B cell subsets in healthy children: Reference values for evaluation of B cell maturation process in peripheral blood. Cytometry 2010;78B(6):372-381

**Table S4** Top ten clones and their amino acid sequencies

|  | **Control 1** | **Control 2** | **Patient** |
| --- | --- | --- | --- |
| 1 | CALWEIELGKKIKVF  (0.2457) | CALWEPQELGKKIKVF (0.1672) | CALWEVELGKKIKVF (0.4018) |
| 2 | CALWLELGKKIKVF  (0.1181) | CALWGMPQELGKKIKVF (0.1223) | CALWESELGKKIKVF (0.3742) |
| 3 | CATWDRQYYKKLF  (0.1144) | CAAWEPRDTGWFKIF (0.0877) | CALWEVGGPQLGKKIKVF (0.0655) |
| 4 | **CALWEVQELGKKIKVF**  (0.06019) | **CALWEVQELGKKIKVF** (0.0654) | CALWEVTELGKKIKVF (0.0345) |
| 5 | CALWEGAKKLF  (0.0288) | CALWEVRELGKKIKVF (0.0534) | CALWEVEKLGKKIKVF (0.0210) |
| 6 | CALWERPELGKKIKVF  (0.0242) | CAAWDWEALPNYYKKLF (0.0392) | CALWEVHQELGKKIKVF (0.0177) |
| 7 | CALWEPTSEQELGKKIKVF  (0.0226) | CALWEDQELGKKIKVF (0.0327) | CATWDGWYYYKKLF (0.0124) |
| 8 | CATWEGYTTGWFKIF  (0.0166) | CALWAELGKKIKVF (0.0321) | CALWDHQAELGKKIKVF (0.0100) |
| 9 | CALWEVRELGKKIKVF  (0.01267) | CALWEKQELGKKIKVF (0.0259) | CALWEVGQELGKKIKVF (0.0078) |
| 10 | CAFNTGWFKIF  (0.0113) | CALWEVKELGKKIKVF (0.0234) | CALWEVPPQELGKKIKVF (0.0033) |

The top ten arrangements and their amino acid sequences, frequencies in parentheses. The most common public clonotype according to literature [15–17] in bold (**CALWEVQELGKKIKVF**).

**Table S5** Overview TCRvγ repertoire sequencing

|  | **Total templates (sum)**^a^ | **Total productive templates (sum)^b^** | **Fraction productive^c^** | **Productive rearrangements^d^** | **Productive Simpson Clonality (within the sample)^e^** |
| --- | --- | --- | --- | --- | --- |
| Control 1 | 621,608 | 506,534 | 0.8149 | 80,326 | 0.2434 |
| Control 2 | 478, 696 | 384,667 | 0.8039 | 43,384 | 0.1999 |
| Patient | 894,729 | 604,180 | 0.6753 | 43,324 | 0.4311 |

^a^Total Templates (Sum) in the sum of template counts for all productive and unproductive rearrangements in the sample.

^b^Total Productive Templates (Sum) is the sum of template counts for all productive rearrangements in the sample.

^c^Fraction productive is the fraction of Productive Templates among all templates.

^d^Productive Rearrangements is the count of unique rearrangements in the sample that are in-frame and do not contain a stop codon. Productive rearrangements can produce a functional protein receptor.

^e^Productive Simpson Clonality is calculated for a sample as the square root of Simpson’s diversity index for all productive rearrangements. Values for clonality range from 0 to 1. Values near 1 represent samples with one or a few predominant rearrangements (monoclonal or oligoclonal samples) dominating the observed repertoire. Clonality values near 0 represent more polyclonal samples.

**Table S6** Pathogenicity predictions of the c.534C>A; p.(Phe178Leu) somatic variant

| **Prediction tool** | **Score** | **Prediction** |
| --- | --- | --- |
| SIFT | 0.64 | Tolerated |
| Polyphen 2 | 0.013 | Benign |
| CADD Score (PHRED) | 15.88 | - * |
| Mutation taster | - (score not provided) | Polymorphism (i.e. probably harmless) |

*There is no clear cut-off for CADD score interpretation. Scores above 20 are predicted to be among the 1.0% most deleterious possible substitutions to be caused in the human genome. However, often pathogenicity predictions made by bioinformatic tools like genome browsers (e.g. Ensembl, USCS) consider CADD scores under 20 as benign or tolerated.

**Table S7** DynaMut2 predictions

| **Structure** | **Variant** | **Predicted Stability**  **Change (ΔΔG)*** | **Prediction** |
| --- | --- | --- | --- |
| 2B5I_WT | Pro58Ser | -1.69 kcal/mol | Destabilizing |
| 2B5I_WT | Phe178Leu | 0.17 kcal/mol | Stabilizing |
| 2B5I_WT | Pro58Ser/Phe178Leu | -0.35 kcal/mol | Destabilizing |

*The change in the Gibbs free energy (ΔΔG) of folding [18,19] calculated as ΔG_WT_-ΔG_mutant_; ΔG=Gibbs free energy


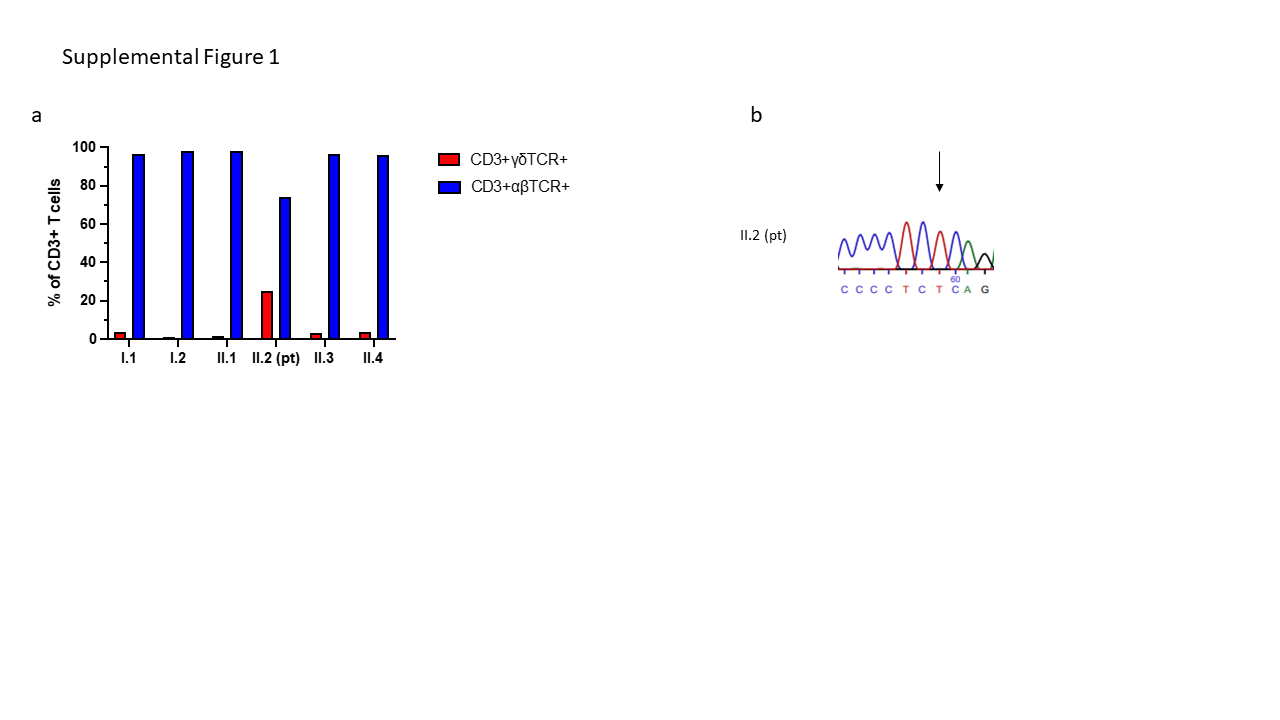


**Fig. S1** A) Patient’s and family members’ γδ (red) and αβ (blue) T cells percentages (of CD3+ T cells) in peripheral blood. B) Patient’s γδ T cells harbour c.172C>T;p.(Pro58Ser) variant (marked with an arrow)


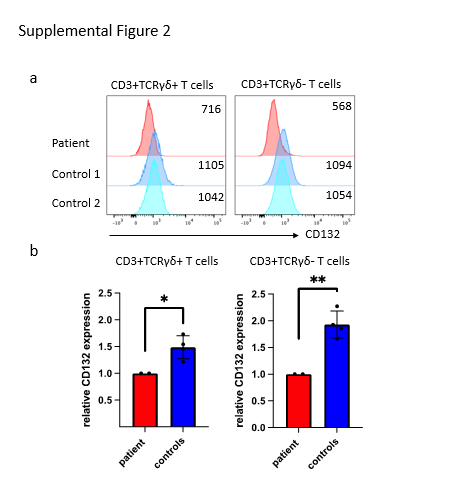


**Fig. S2** A) IL2RG (CD132) total cell associated protein expression in patient’s and healthy controls’ CD3+γδ+ (left) and CD3+γδ- (right) T cells (patient red, controls blue) measured as MFI. B) Relative expression of IL2RG (CD132) in CD3+γδ+ (left) and CD3+γδ- (right) T cells, combined from two independent experiments. Patient MFI normalized as one in b, error bars represent SD. * = p < 0.05; ** = p < 0.01; determined by unpaired t-test with Welch’s correction


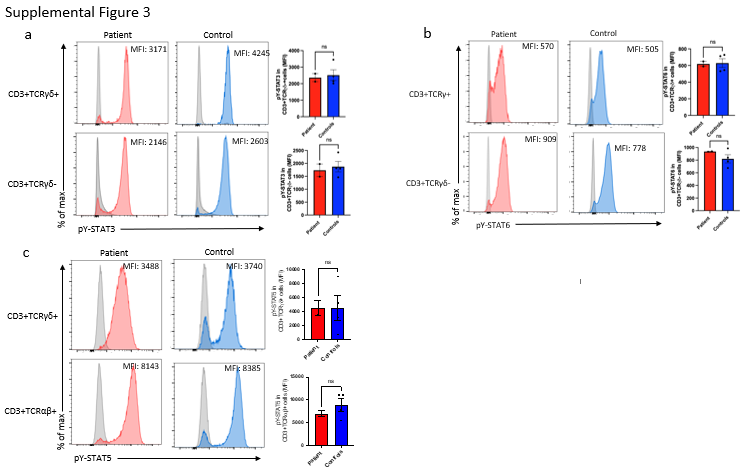


**Fig.S3** (A) STAT3 phosphorylation in response to 50ng/ml IL-21 and (B) STAT6 phosphorylation in response to 100ng/ml IL-4 in patient’s and healthy controls’ CD3+γδ+ and CD3+γδ- cells. (C) STAT5 phosphorylation in response to 50 ng/ml IL-7 in patient’s and healthy controls’ CD3+γδ+ and CD3+αβ+ cells. Data is representative of two independent experiments. Cumulative bar graphs (for healthy controls n=2 in each repeat, difference in MFI determined as stimulated - unstimulated); ns=non-significant determined by unpaired t-test with Welch’s correction, error bars indicate SEM.


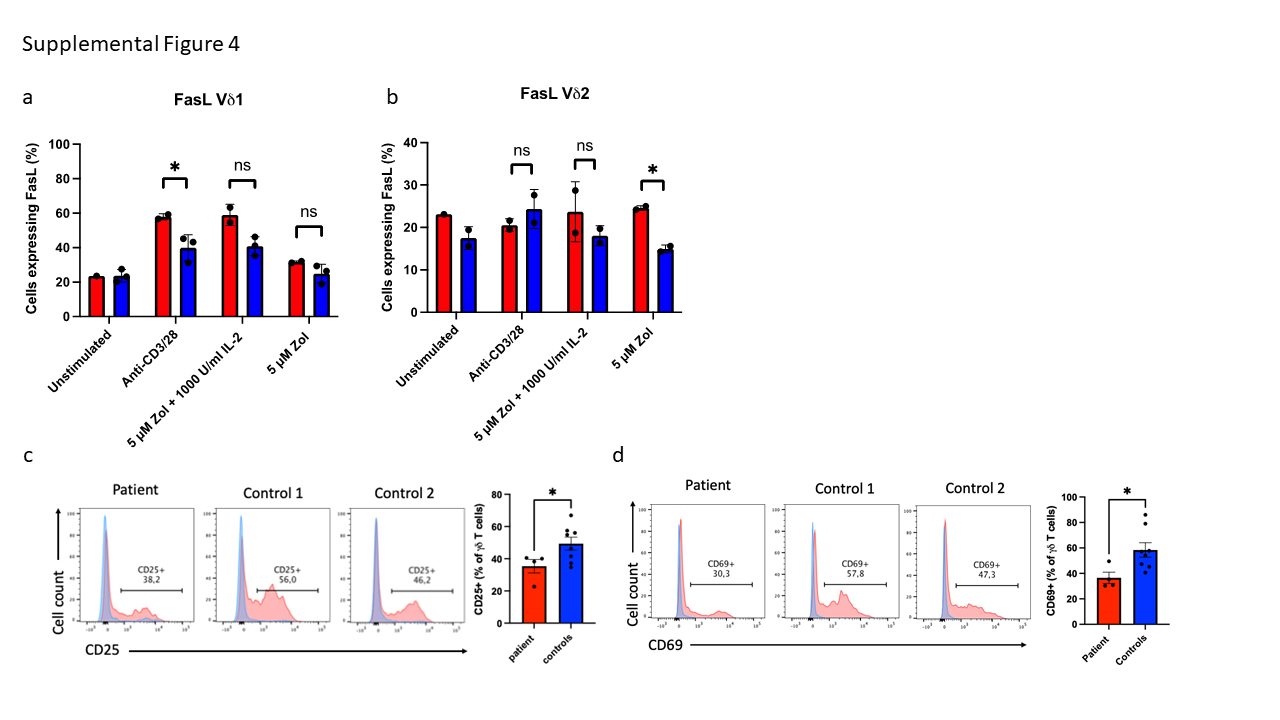


**Fig.S4** Additional phenotyping data. Fas-ligand (FasL) expression in patient’s and healthy controls’ (A) Vδ1 and (B) Vδ2 cells. Patient in red, controls in blue. Expression of (C) CD25 and (D) CD69 in patient’s and healthy controls’ γδ T cells after 24h anti-CD3/28 stimulation. Blue trace unstimulated, red trace stimulated. * = p < 0.05, ns=non-significant determined by unpaired t-test with Welch’s correction


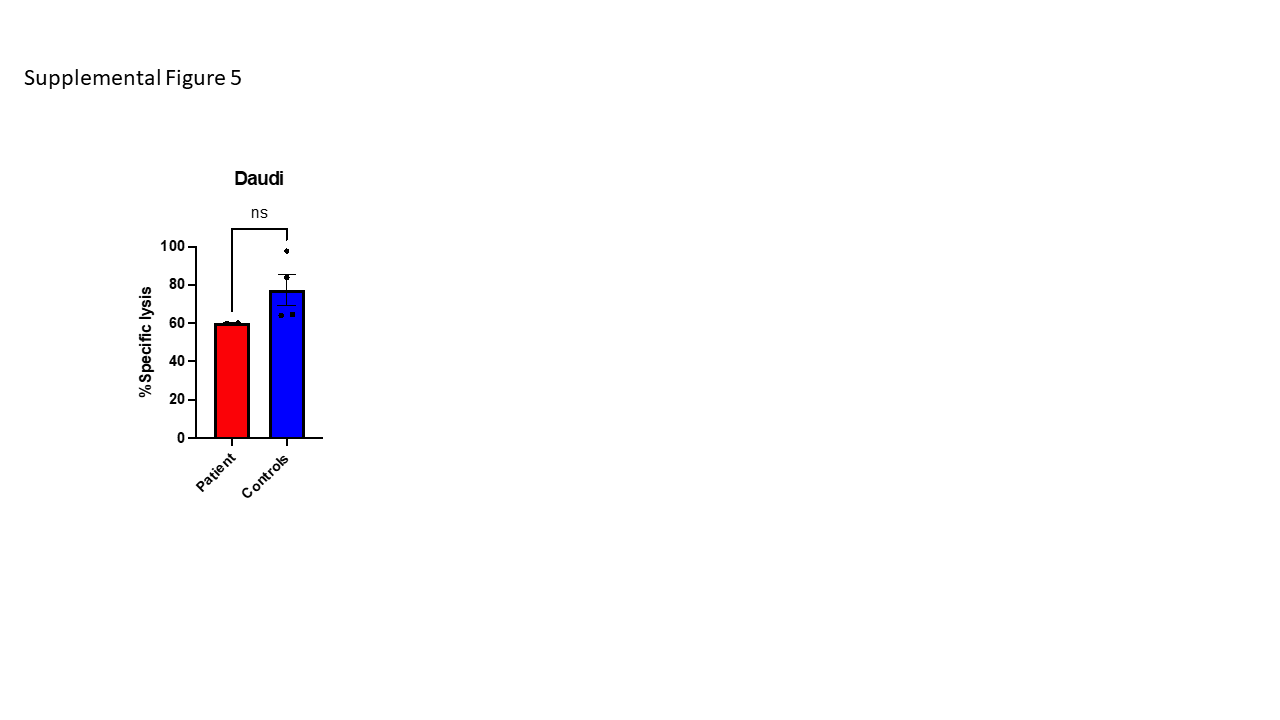


**Fig.S5** Killing of Daudi cells by isolated and non-activated γδ T cells. Cumulative data from two independent experiments, each dot represents average of two to three technical replicates from patient and four independent controls; error bars SEM, ns=non-significant determined by unpaired-t-test with Welch’s correction

**
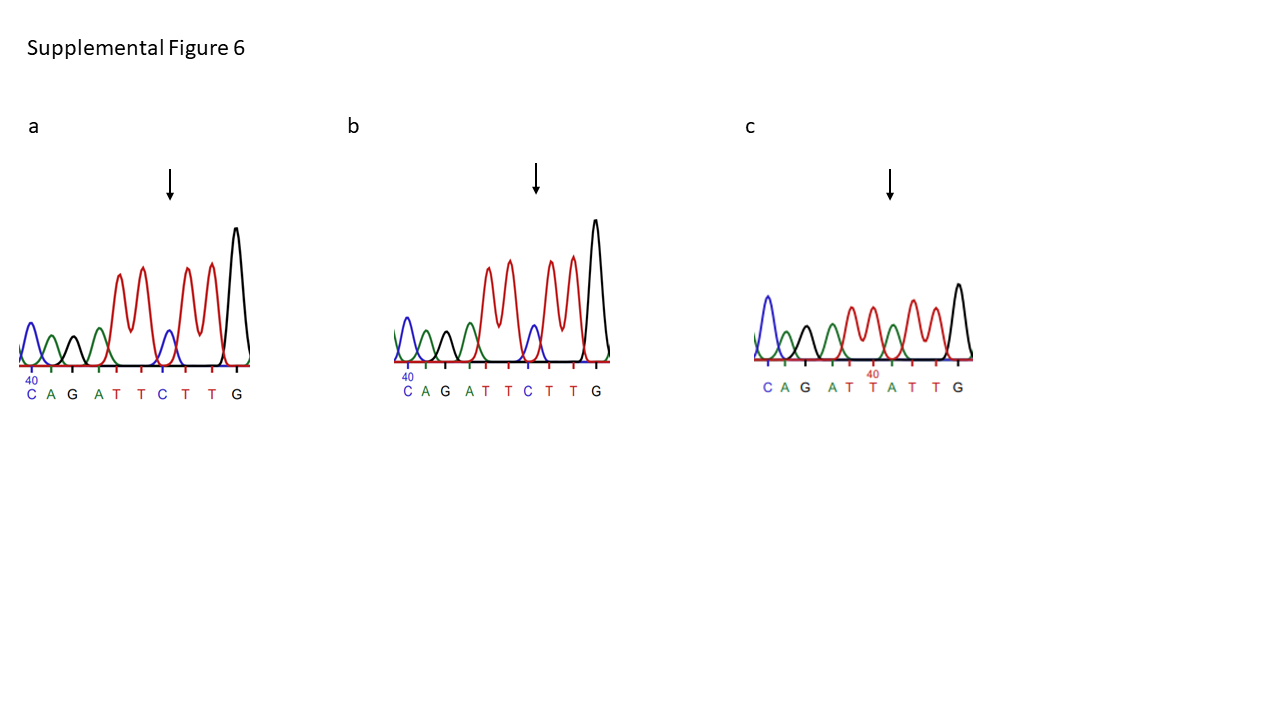
**

**Fig.S6** Sanger sequencing of *IL2RG* gDNA in patient’s (A) αβ (B) CD19+ and (C) γδ T cells (after 10-d *in vitro* expansion). Somatic c.534C>A; p.(Phe178Leu) variant present in γδ T cells, position of the mutated base marked with an arrow

**References**

[1] Tuovinen EA, Grönholm J, Öhman T, Pöysti S, Toivonen R, Kreutzman A, et al. Novel Hemizygous IL2RG p.(Pro58Ser) Mutation Impairs IL-2 Receptor Complex Expression on Lymphocytes Causing X-Linked Combined Immunodeficiency. J Clin Immunol 2020;40:503–14. https://doi.org/10.1007/s10875-020-00745-2.

[2] Kondo M, Izumi T, Fujieda N, Kondo A, Morishita T, Matsushita H, et al. Expansion of human peripheral blood γδ T cells using zoledronate. J Vis Exp JoVE 2011:3182. https://doi.org/10.3791/3182.

[3] Carlson CS, Emerson RO, Sherwood AM, Desmarais C, Chung M-W, Parsons JM, et al. Using synthetic templates to design an unbiased multiplex PCR assay. Nat Commun 2013;4:2680. https://doi.org/10.1038/ncomms3680.

[4] Robins HS, Campregher PV, Srivastava SK, Wacher A, Turtle CJ, Kahsai O, et al. Comprehensive assessment of T-cell receptor β-chain diversity in αβ T cells. Blood 2009;114:4099–107. https://doi.org/10.1182/blood-2009-04-217604.

[5] Marits P, Wikström A-C, Popadic D, Winqvist O, Thunberg S. Evaluation of T and B lymphocyte function in clinical practice using a flow cytometry based proliferation assay. Clin Immunol 2014;153:332–42. https://doi.org/10.1016/j.clim.2014.05.010.

[6] Rodrigues CHM, Pires DEV, Ascher DB. DynaMut2: Assessing changes in stability and flexibility upon single and multiple point missense mutations. Protein Sci 2021;30:60–9. https://doi.org/10.1002/pro.3942.

[7] Liu X, Salokas K, Tamene F, Jiu Y, Weldatsadik RG, Öhman T, et al. An AP-MS- and BioID-compatible MAC-tag enables comprehensive mapping of protein interactions and subcellular localizations. Nat Commun 2018;9:1188; 1188–1188. https://doi.org/10.1038/s41467-018-03523-2.

[8] Liu X, Salokas K, Weldatsadik RG, Gawriyski L, Varjosalo M. Combined proximity labeling and affinity purification−mass spectrometry workflow for mapping and visualizing protein interaction networks. Nat Protoc 2020;15:3182–211. https://doi.org/10.1038/s41596-020-0365-x.

[9] Meier F, Brunner A-D, Koch S, Koch H, Lubeck M, Krause M, et al. Online Parallel Accumulation–Serial Fragmentation (PASEF) with a Novel Trapped Ion Mobility Mass Spectrometer *. Mol Cell Proteomics 2018;17:2534–45. https://doi.org/10.1074/mcp.TIR118.000900.

[10] Yu F, Haynes SE, Teo GC, Avtonomov DM, Polasky DA, Nesvizhskii AI. Fast Quantitative Analysis of timsTOF PASEF Data with MSFragger and IonQuant. Mol Cell Proteomics 2020;19:1575–85. https://doi.org/10.1074/mcp.TIR120.002048.

[11] Kong AT, Leprevost FV, Avtonomov DM, Mellacheruvu D, Nesvizhskii AI. MSFragger: ultrafast and comprehensive peptide identification in mass spectrometry–based proteomics. Nat Methods 2017;14:513–20. https://doi.org/10.1038/nmeth.4256.

[12] Teo GC, Polasky DA, Yu F, Nesvizhskii AI. Fast Deisotoping Algorithm and Its Implementation in the MSFragger Search Engine. J Proteome Res 2021;20:498–505. https://doi.org/10.1021/acs.jproteome.0c00544.

[13] Teo G, Liu G, Zhang J, Nesvizhskii AI, Gingras A-C, Choi H. SAINTexpress: Improvements and additional features in Significance Analysis of INTeractome software. Spec Issue Can Proteomics Fill Gap Genomics Phenotypes 2014;100:37–43. https://doi.org/10.1016/j.jprot.2013.10.023.

[14] Mellacheruvu D, Wright Z, Couzens AL, Lambert J-P, St-Denis NA, Li T, et al. The CRAPome: a contaminant repository for affinity purification–mass spectrometry data. Nat Methods 2013;10:730–6. https://doi.org/10.1038/nmeth.2557.

[15] Willcox CR, Davey MS, Willcox BE. Development and Selection of the Human Vγ9Vδ2+ T-Cell Repertoire. Front Immunol 2018;9:1501. https://doi.org/10.3389/fimmu.2018.01501.

[16] Sherwood Anna M., Desmarais Cindy, Livingston Robert J., Andriesen Jessica, Haussler Maximilian, Carlson Christopher S., et al. Deep Sequencing of the Human TCRγ and TCRβ Repertoires Suggests that TCRβ Rearranges After αβ and γδ T Cell Commitment. Sci Transl Med 2011;3:90ra61-90ra61. https://doi.org/10.1126/scitranslmed.3002536.

[17] Davey MS, Willcox CR, Joyce SP, Ladell K, Kasatskaya SA, Mclaren JE, et al. Clonal selection in the human V[delta]1 T cell repertoire indicates [gamma][delta] TCR-dependent adaptive immune surveillance. Nat Commun 2017;8:14760. https://doi.org/10.1038/ncomms14760.

[18] Pires DEV, Ascher DB, Blundell TL. mCSM: predicting the effects of mutations in proteins using graph-based signatures. Bioinformatics 2014;30:335–42. https://doi.org/10.1093/bioinformatics/btt691.

[19] Pires DEV, Chen J, Blundell TL, Ascher DB. In silico functional dissection of saturation mutagenesis: Interpreting the relationship between phenotypes and changes in protein stability, interactions and activity. Sci Rep 2016;6:19848. https://doi.org/10.1038/srep19848.
